# Factors Affecting Breastfeeding Practices under Lockdown during the COVID-19 Pandemic in Thailand: A Cross-Sectional Survey

**DOI:** 10.3390/ijerph18168729

**Published:** 2021-08-18

**Authors:** Chanodom Piankusol, Wachiranun Sirikul, Krongporn Ongprasert, Penprapa Siviroj

**Affiliations:** Department of Community Medicine, Faculty of Medicine, Chiang Mai University, Chiang Mai 50200, Thailand; chanodom.p@cmu.ac.th (C.P.); wachiranun.sir@cmu.ac.th (W.S.); krongporn.o@cmu.ac.th (K.O.)

**Keywords:** COVID-19, lockdown, breastfeeding, infant feeding support, health care service contact, Thailand

## Abstract

A COVID-19 lockdown and restrictive order has had a large impact on the lives of people. This cross-sectional study was conducted to identify factors affecting breastfeeding among mothers living in Thailand during the lockdown. Data were collected from 903 mothers with infants ages 0–12 months from 17 July 2020 to 17 October 2020 after the first nationwide COVID-19 lockdown period by an online platform and interview questionnaire survey. Multivariable logistic regression analysis was used to investigate the association between the effect of lockdown and breastfeeding practices with potential confounder adjustment including maternal age, ethnicity, newborn age <6 months, family income below $16,130 per annum, education below undergraduate level, and working status. Mothers changed breastfeeding practices in this period (*n* = 39, 4.32%) including having changed from exclusive breastfeeding to combined breastfeeding with formula milk (*n* = 22, 2.44%), and having reduced the frequency when compared to before the pandemic (*n* = 13, 1.44%). The associated factors of changing breastfeeding practices were “contact with healthcare services” (aOR = 0.46, 95% CI 0.22 to 0.96, *p* = 0.04), “infant feeding support from health personnel” (aOR = 0.39, 95% CI 0.16 to 1.94, *p* = 0.035), and “lack family support and help with feeding your baby after lockdown” (aOR = 7.04, 95% CI 1.92 to 25.84, *p* = 0.003). In conclusion, this study showed a slight decrease in breastfeeding in the sampled mothers during the COVID-19 lockdown in Thailand. A long-term national surveillance system for maintenance of breastfeeding should be established. Health care service interventions and additional information are needed to support mothers and families for breastfeeding during pandemics.

## 1. Introduction

The coronavirus disease 2019 (COVID-19) is a worldwide crisis and has severely impacted all countries, with more than 179 million confirmed cases as of 25 June 2021, including 3,899,172 deaths [1]. COVID-19 indicates the magnitude of severe acute respiratory syndrome-related coronavirus (SARS-CoV) and pandemic influenza, indicating a risk of global spread. The possible main route of transmission is thought to be close contact and respiratory droplets [2,3]. Social distancing is the practice of increasing the space between people to decrease the chance of spreading illness [4]. All individuals can take part in reducing the risk of infection and have the potential to slow the rate of infection during this pandemic. Therefore, social distancing via lockdown or stay-at-home order has been the most restrictive policy and is a preventive measure to protect healthy people from infection and is highly important to control COVID-19 [5,6,7].

Lockdowns are an effective pandemic response strategy; however, maintaining physical distance can impact people’s lives in various aspects including reduced access to health services, social, economic, and health consequences. For example, under COVID-19 lockdown, screenings and diagnostics for chronic health issues have been delayed and may cause a delay in treatment or worsening consequences [8,9,10]. The association between mental health and well-being during the COVID-19 pandemic has been widely investigated. The lockdowns substantially increase depressive thoughts, anxiety, stress, suicide, and other issues [11,12]. These negative influences could be due to lifestyle disruptions [13,14], and the magnitude of these problems is varies between different groups of people. Two earlier studies in the UK showed that mental health and well-being among young adults and women was worse during the COVID-19 pandemic than other groups [9,15]. Moreover, family life in lockdown has a high percentage of reported disruptions in family life patterns, chore allocations, household tensions, and domestic responsibilities for mothers, who were already carrying out most of the household labor [16,17]. The findings of the previous study observed that the impact of the lockdown situation was different between groups [9,15,16,17]. Women and children are vulnerable populations, especially lactating mothers and newborns who need special support from their families and health care systems to succeed in breastfeeding.

The World Health Organization recommends early initiation of breastfeeding and exclusive breastfeeding during the first six months of life and continued breastfeeding until 24 months of life [18]. Although breastfeeding provides benefits to infants and mothers, multiple factors are involved in the decision to initiate and continue breastfeeding. The literature shows that the factors influencing mothers’ decision to breastfeed are sociocultural, economic, environmental, and personal, whereas the lockdown situation has affected those factors. Other studies also found that further factors, e.g., socioeconomic status, physical-psychological status, social support, the level of health service support related to information and communication systems, and the protection of maternal well-being, have a positive or negative effect on breastfeeding [19,20,21]. The mother’s decision to not breastfeed has major long-term effects on the health, nutrition, and development of the child and mother’s health [22,23]. Many barriers to breastfeeding exist at the societal rather than individual level [24]. Currently, mothers were particularly affected by the COVID-19 lockdown order. They had to adapt rapidly to changes and uncertain situations, deal with misinformation about the safety of breastfeeding, and could not breastfeed symptomatic babies [25,26]. Mothers in the UK (13%) reported a change in feeding often related to a lack of breastfeeding support [27]. Previous studies reported a negative impact from lack of social and emotional support on the maternal breastfeeding experience. The most common reason to stop breastfeeding was insufficient professional support and lack of face-to-face support (70.3%). The important sources of feeding support were the partner (60%), health professional (50%) and online groups (47%) [27,28].

In Thailand, on 13 January 2020, the government reported the first confirmed case of COVID-19, which continued to spread. The Thai government imposed the first nationwide curfew for all people from 10 p.m. to 4 a.m., and lockdown from 3 April to 15 June 2020 [29]. As of 24 June 2021, there were 232,647 cases of COVID-19 in Thailand and 1775 deaths [30]. Several studies reported in some countries the impact of the COVID-19 lockdown as a disruption of lifestyle, family life, and behavior, especially breastfeeding. However, no data have been reported in Thailand. This study aimed to identify factors affecting breastfeeding practices among mothers living in Thailand during the lockdown in the COVID-19 pandemic. 

## 2. Materials and Methods

### 2.1. Study Setting and Design

**Figure 1:**
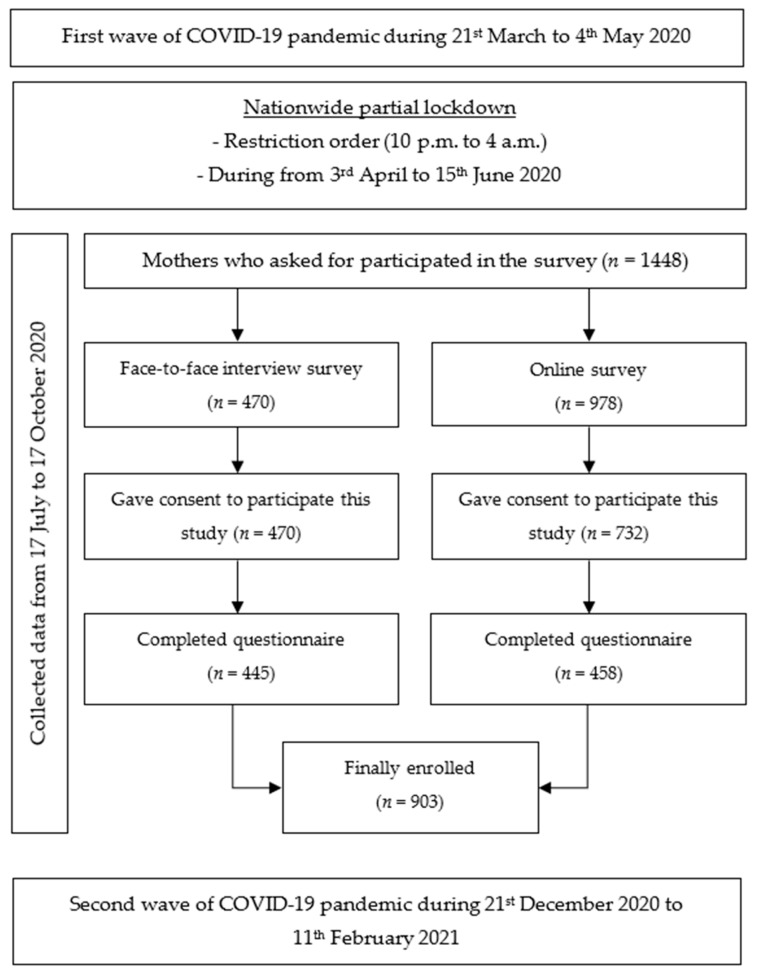
COVID-19 pandemic and lockdown durations in Thailand and period of the online platforms, interviews and sample size of mothers who were willing to participate in the survey.

This cross-sectional study involved the recruitment of 903 women over 18 years old with an infant under 12 months of age at the time of the survey, regardless of nationality, living in Thailand during the COVID-19 pandemic. In this study, the nationwide online survey and interview questionnaire in Chiang Mai Province survey was conducted from 17 July 2020 to 17 October 2020 after the first nationwide COVID-19 lockdown period in Thailand (from 3 April to 15 June 2020) and again later—from 21 December 2020 to 11 February 2021 (Figure 1). The participants were asked retrospectively about the effects of the COVID-19 pandemic on breastfeeding practices during the lockdown. 

The participants were invited via social media to answer a one-time online survey conducted by the Department of Community Medicine, Faculty of Medicine, Chiang Mai University, Thailand. The participants filled out the questionnaire using a cell phone or laptop/computer with internet access. Another group participated in self-completed face-to-face interviews during visits to the well-baby clinic at four hospitals (public and private sectors) in Chiang Mai Province. These hospitals represent primary care (Sankampaeng Health-Promoting Hospital), secondary care (Sansai District Hospital and Health Promotion Hospital at the Regional Health Promotion Center 1), and tertiary care (Sriphat Medical Center, a private hospital). All the participants were anonymous, with no recorded identification. Free copies of the document and informed consent were available on the face-to-face study site. 

For data collection and management, the Research Electronic Data Capture (REDCap) platform (https://redcap.med.cmu.ac.th/surveys/index.php?s=7Y49XT4A78 access on 17 July 2020 to 17 October 2020) was used in both nationwide online platforms and interviews. The sample size of this study was to provide as many responses as possible within the time frame to maximize the usefulness of the results. For the online survey, a total of 978 participants accessed the online platform, and 732 participants (74.85%) gave their consent to participate in this study. Of those, 458 participants (46.83%) answered complete questions from the nationwide online platform. In contrast, a face-to-face interview survey had 445 (94.68%) participants of 470 who answered complete questions. Thus, the sample population in this study was 903 participants consisting of nationwide online (458, 50.72%) and face-to-face interviews (445, 49.28%).

### 2.2. Questionnaire Design

This study used a structured questionnaire that was developed by experts in Pediatrics, Child Nutrition and Dietetics and Anthropology, University College London (UCL) in English, translated into Thai, adapted for the Thai context, and pilot questionnaire tested before dissemination. 

The questionnaire had the following three parts:

(1) Socio-demographic characteristics—maternal age and ethnicity; maternal education; working status before labor; current working status; household income per year; type of housing, e.g., own house, apartment/condominium, dormitory, and rental house; family composition; infant age; and gender.
(2) Infant feeding practices and changes during COVID-19 lockdown—the questions in this section asked about how mothers have changed breastfeeding practices including having changed from exclusive breastfeeding to combined breastfeeding with formula milk, and having reduced the frequency when compared to before the pandemic and stopped breastfeeding. The answers to these questions are “yes” or “no”.
(3) Perceived effects of COVID-19 lockdown—the questions in this section asked about how the lockdown impacted maternal lives and activities in both positive and negative ways, and perceived support during COVID-19. Details are as follows:
  (3.1) The frequency of different activities such as leaving the home for work, exercise, grocery shopping, and engaging in online social activities (every day or more per week, 4–5 times per week, 1–3 times per week, never).
  (3.2) Receiving infant feeding support from the family and couple after lockdown.
  (3.3) Maternal perception about family impacts from the lockdown, household crowding after lockdown, and family stress from confinement (mostly, often, sometimes, not at all, 4-point Likert scale).
  (3.4) The frequency of access to contact with health care services and infant feeding support from health personnel (every day or more per week, 4–5 times per week, 1–3 times per week, never).
  (3.5) The frequency of access to infant feeding and childcare support from infant feeding support groups (every day or more per week, 4–5 times per week, 1–3 times per week, never).

### 2.3. Statistical Analyses

All statistical analysis was performed via the STATA software package (Stata Corp. 2019. Stata Statistical Software: Release 16, Stata Corp LLC, College Station, TX, USA). Derived data from the survey were exported from the REDCap. The participant characteristics were described by the frequency with percentages and mean with a standard deviation for parametric data. The maternal characteristics were compared between mothers with and without change in breastfeeding practices using Fisher’s exact test for categorical data and independent sample t-test for parametric data, and rank-sum test for non-parametric data. The association between changing breastfeeding practices and affecting variables was explored by multivariable logistic regression with potential confounder adjustments including maternal age, ethnicity, newborn age < 6 months, family income below USD 16,130, education below undergraduate level, and working status. The results of this study were analyzed according to the Strengthening of the Reporting of Observational Studies in Epidemiology (STROBE) checklist. All statistical analyses were two-sided, and a *p*-value < 0.05 was considered statistically significant.

### 2.4. Ethical Consideration

This study was conducted following the Declaration of Helsinki and the protocol was approved by the Research Ethics Committee, Faculty of Medicine, Chiang Mai University, Thailand (Study Code: COM-2563-07416). 

## 3. Results

### 3.1. Sample Characteristics and Changing Breastfeeding Practices

The characteristics of the study sample are presented in Table 1. The sample comprised 903 women with a mean age of 31.40 years (SD ± 5.01), and the mean age of the infants was 5.50 (SD ± 3.18) (data not shown). Most subjects were Thai (90.25%), nuclear family (85.38%), working before labor (77.63%), and had their own house (79.96%). Mothers who returned to work were employed (73.32%), business owners (15.26%), and unemployed (11.41%). Two-thirds of participants (69.55%) received education below bachelor’s degree. Almost all (*n* = 864, 95.68%) have not changed breastfeeding practices during the COVID-19 lockdown. Only 4.32% of sampled mothers (*n* = 39) have changed breastfeeding practices, including having changed from exclusive breastfeeding to combined breastfeeding with formula milk (*n* = 22, 2.44%), reduced the frequency when compared to before the pandemic (*n* = 13, 1.44%), and stopped breastfeeding (*n* = 4, 0.44%) (data not shown). There were no significant differences in distributions of characteristics between mothers who had changed and not changed breastfeeding practices during the COVID-19 lockdown (see Table 1). 

### 3.2. The Association between Recreational Activities, Perceived Effect of Lockdown, and Changing Breastfeeding Practices

The recreational activities and effects of the COVID-19 lockdown associated with mothers who changed breastfeeding practices are presented in Table 2. Between mothers who had changed and not changed breastfeeding practices, the mothers who had changed breastfeeding practices have more online activities (74.36% and 60.88%, respectively) and outdoor activities (69.23% and 64.81%, respectively) than those who had not changed breastfeeding practices without significant differences (*p* = 0.091 and *p* = 0.572, respectively). For the perceived effect of lockdown, the results found that mothers who changed breastfeeding practices had reported the family impacts “household crowding after lockdown” and “family stress from confinement” more than those who had not changed (*p* = 0.015 and *p* = 0.010, respectively). Regarding family support, mothers who changed breastfeeding practices reported that they “lack family support and help with feeding your baby after lockdown” more than those who had not changed (*p* < 0.001). Concerning health care support, the mothers who changed breastfeeding practices reported to have “received infant feeding support from health personnel”, “enough maternal health support” and “overall support satisfaction”. In terms of contact for consultation, those who changed breastfeeding practices had less “contact with healthcare services” than those who had not changed (*p* < 0.001, *p* < 0.001 and *p* = 0.003, respectively). 

### 3.3. The Associated Factors of Changing Breastfeeding Practices

The associated factors of changing breastfeeding practices are presented in Table 3. Logistic regression analysis adjusted odds ratio showed the preventive variables, perceived effect of lockdown including “contact with healthcare services” and “infant feeding support from health personnel” of mothers who changed breastfeeding practices had 0.46 times (aOR 0.46, 95% CI 0.22 to 0.96, *p* = 0.040) and 0.39 times (aOR 0.39, 95% CI 0.16 to 0.94, *p* = 0.035), respectively. The adjusted ORs were lower than 1 when compared to those who did not change during the COVID-19 pandemic, while mothers who changed breastfeeding had 7.04 times higher odds of “lack family support and help with feeding your baby after lockdown” than those who did not change (aOR 7.04, 95% CI 1.92 to 25.84, *p* = 0.003). Thus, “lack family support and help with feeding your baby after lockdown” was the risk factor that significantly impacted changing breastfeeding practices. 

## 4. Discussion

The COVID-19 lockdown restrictions are unprecedented and have disrupted people’s lifestyles in Thailand. This study indicates that the influence of lockdown on breastfeeding in mothers who have an infant aged 0–12 months has resulted in a slight decrease in breastfeeding (4.32%), especially leading to mother’s changing the method, reducing the frequency and stopping breastfeeding. Moreover, our findings showed that the predictors of breastfeeding practices as preventive factors including “contact with healthcare services” and “health personnel support” were positively associated, and a risk factor of breastfeeding practices during lockdown restrictions was “lack family support for infant feeding”. 

Our study revealed that breastfeeding practices of most participants during the COVID-19 lockdown remained similar to the patterns before lockdown. These findings contrasted with studies in Italy and the UK; lockdown and home confinement led to a decrease in exclusive breastfeeding [31], and 10% of mothers reported a decrease in breastfeeding frequency and 15% a decrease in feed duration [27]. These inconsistent results might be due to the differences in social awareness and mass communication regarding the COVID-19 situation in each country. In the region with more serious situations, the population may have a higher fear of contracting the COVID-19 virus. This may cause lifestyle disruption and the decision to reduce or stop breastfeeding. Many recommendations suggest that mothers suspected of being infected or infected with COVID-19 should continue breastfeeding with necessary respiratory hygiene precautions during feeding [32,33,34]. However, an uncertain situation from the first wave of the COVID-19 pandemic led to misinformation about the safety of breastfeeding and inability to breastfeed if the mother had symptoms of COVID-19 [25,26]. It is difficult to compare our results due to the COVID-19 pandemic in Thailand at that time because Thailand had fewer confirmed cases and deaths as well as shorter lockdown periods [29]. Thai residents were instructed to remain inside their homes between 10 p.m. to 4 a.m. except for essential activities, e.g., medical care and purchasing food [29], which did not directly affect contact with health care services and support by health personnel. 

A mother’s decision to breastfeed or formula feed is affected by a range of factors. Professional support can encourage or help mothers who want to overcome problems related to breastfeeding [35]. Our study reported supporting infant feeding by health personnel and accessing health services for consultation were positively associated with breastfeeding practices during the COVID-19 pandemic. When comparing this to a survey conducted with mothers in the UK, a similar factor to health professional support associated with breastfeeding was reported during the lockdown [27]. An online survey in the UK reported that the COVID-19 affected parents, babies, and the services that support them. There are differences in lockdown experience, received care and support, and accessing information [36]. Moreover, in our findings, considering mothers who changed breastfeeding had 7.04 times higher odds of “lack family support and help with feeding your baby after lockdown” than those who did not change. Family and paternal supports are important both physically and emotionally and potentially affect the decision to not breastfeed [37]. Previous studies reported that maternal grandmother, partner breastfeeding support, and exclusive breastfeeding duration were associated with breastfeeding maintenance for at least 12 months [38,39,40,41]. The professional support system offers positive counseling and breastfeeding and affects decisions to breastfeed [41]. Disruption to healthcare from the COVID-19 pandemic and lockdown have resulted in a loss of health service contact and lack support from health professionals. Some of the healthcare supports moved to online or were canceled [5,8]. The COVID-19 pandemic disrupted lifestyles and may have reduced support from family members due to social distancing [40]. Regarding, disrupted lifestyles from the COVID-19 pandemic and lockdown, supports from family members have decreased due to social distancing. Mothers felt isolated, and this might lead to mental health problems such as depressive and anxiety symptoms and loneliness, which consequently affects mothers’ decision not to breastfeed [13,14,15,17,27,42]. 

Unlike other studies [19,20,21], this study did not find an association between breastfeeding practices and other support groups, such as the mothers’ group. Nor did we find the association of psychological problems from COVID-19 pandemic, e.g., household crowding after lockdown, family stress from confinement, and limitation of activities with breastfeeding practices. Since data collection was carried out in the period of the first lockdown in Thailand, when the COVID-19 was well controlled, and with the lockdown being a short period of time, these may not have affected maternal mental health. In addition, this may also be due to little supportive medical data and recommendations regarding breastfeeding, placing restrictions on access to a variety of health information in Thailand regarding breastfeeding during the COVID-19 pandemic. Therefore, long-term studies should be conducted following the outbreak of widespread and severe pandemics. 

To our knowledge, this article is the first to report the result of the impact of the COVID-19 pandemic, lockdown, and restrictive orders on breastfeeding among mothers in Thailand. Our study provided some evidence of slightly decreased breastfeeding practices during the lockdown. Both online and face-to-face interview surveys were utilized to collect data rapidly from a large group of mothers one month after the end of the first lockdown and before the second wave of the COVID-19 pandemic in Thailand. However, face-to-face interviews of our study were collected only in Northern Thailand, which took place at secondary and tertiary public hospitals, and private hospitals to cover various maternal socioeconomic statuses. On the other hand, the online survey collected data from all regions of Thailand. Therefore, the online and face-to-face surveys proved effective. Another limitation of this study was part of the sample having an online survey with an inherent selection bias not representative of all mothers with an infant of 0–12 months in Thailand. The sample size of the online and face-to-face interview survey of this study was small and limited; therefore, detailed subgroup analysis of online and interview surveys was not possible. Lastly, mothers who did not have access to the internet by either cell phone or computer were not able to participate in the survey. Finally, the bias in causal associations with cross-sectional studies cannot be disregarded. 

## 5. Conclusions

In conclusion, this study reported that the social distancing as a result of the lockdown or stay-at-home order of the COVID-19 pandemic resulted in slightly decreased breastfeeding practices in sampled mothers with an infant aged 0–12 months during lockdown in Thailand. The predictive indicators of preventive variables were “contact with healthcare services” and “feeding support from health personnel”, which are strongly positively associated with affecting breastfeeding practices. Additionally, the lack of family support for infant feeding is the risk factor that significantly impacts changing breastfeeding practices. To combat reductions in or halting of breastfeeding in the lockdown situation due to the COVID-19 pandemic, a long-term national surveillance system should be established. Health care services need to intervene to support mothers to initiate and continue breastfeeding. Family support for infant feeding should be promoted, and additional information and public relations throughout the COVID-19 pandemic should be provided. Additionally, further research must explore the effects of lockdown on various dimensions of the maternal experience, maternal mental health, and barrier variables associated with exclusive and continued breastfeeding. 

## Figures and Tables

**Table 1:** Comparison of characteristics between mothers who had changed and not changed breastfeeding practices during the COVID-19 lockdown (*n* = 903).

Characteristics	Total (*n* = 903)		Changed Breastfeeding Practices ** (*n* = 39, 4.32%)		No Change in Breastfeeding Practices (*n* = 864, 95.68%)		*p*-Value
	*n*	%	*n*	%	*n*	%	
Maternal age							
≤18 years	2	0.22	-	-	2	0.23	0.591
19–35 years	705	78.07	29	74.36	676	78.24	
>35 years	196	21.71	10	25.64	186	21.53	
Infant age							
≤6 months	572	63.34	19	48.72	553	64.01	0.130
6–12 months	331	36.66	20	51.28	311	35.99	
Infant gender							
Male	485	53.70	20	51.28	465	53.82	0.756
Female	418	46.30	19	48.72	399	46.18	
Ethnicity							
Thai	815	90.25	37	94.87	778	90.05	0.320
Others *	88	9.75	2	5.13	86	9.95	
Family status							
Single mother	132	14.62	3	7.69	129	14.35	0.166
Nuclear family	771	85.38	36	92.31	735	85.07	
Education							
Below bachelor’s degree	628	69.55	31	79.49	597	69.10	0.213
Bachelor’s degree or above	275	30.45	8	20.51	267	30.90	
Working status before labor							
Working	701	77.63	33	84.62	668	77.31	0.285
Not working/Unemployed	202	22.37	6	15.38	196	22.69	
Current working status (*n* = 701)							
Not working/Unemployed	80	11.41	2	6.06	78	11.82	0.468
Employed	514	73.32	27	81.82	479	72.58	
Business owner	107	15.26	4	12.12	103	15.61	
Household income per year							
Less than USD 16,130	314	34.88	14	35.90	301	34.84	0.866
More than USD 16,130	588	65.12	25	64.10	563	65.16	
Type of housing							
Own house	722	79.96	32	82.05	690	79.86	0.088
Apartment/Condominium	46	5.09	1	2.56	45	5.21	
Dormitory	114	12.62	3	7.69	111	12.85	
Rental house	18	1.99	3	7.69	15	1.74	
Others	3	0.33	0	0.00	3	0.35	

* Others include Burmese, Chinese, Taiwanese, and American; ** Changed breastfeeding practices means changed from exclusive breastfeeding to combined breastfeeding with formula milk, reduced frequency, and stopped breastfeeding. Independent sample *t*-test.

**Table 2:** Comparison of recreational activities and perceived effects of lockdown between mothers who had changed and not changed breastfeeding practices during the COVID-19 lockdown.

Variables	Changed Breastfeeding Practices (*n* = 39)		No Change in Breastfeeding Practices (*n* = 864)		*p*-Value
	*n*	%	*n*	%	
**Recreational activities**					
Online activities	29	74.36	526	60.88	0.091
Outdoor activities	27	69.23	560	64.81	0.572
**Perceived effect of COVID-19 lockdown ^¥^**					
**Family impacts**					
Household crowding after lockdown	11	28.11	122	14.12	0.015 *
Family stress from confinement	4	10.26	24	2.83	0.010 **
**Family support**					
Lack of family support and help with feeding your baby after lockdown	4	10.26	12	1.39	<0.001 **
Infant feeding support in a couple	24	61.54	562	61.54	0.653
**Healthcare and mother’s group support**					
Received infant feeding support from health personnel	30	76.92	805	93.17	<0.001 **
Enough maternal health support	28	71.79	803	92.94	<0.001 **
Overall support satisfaction	24	61.54	802	92.82	<0.001 **
**Contact for consultation**					
Contact with healthcare service	13	33.33	494	57.18	0.003 **
Contact with a “Mother and Baby or breastfeeding support group”	17	43.59	362	41.90	0.834

^¥^ Effect of the COVID-19 lockdown as identified by mothers, * Significant association at *p* < 0.05, ** Significant association at *p* < 0.01.

**Table 3:** Full exploratory model of the associated factors of changing breastfeeding practices using multivariable logistic regression.

Variables in the Exploratory Model	aOR	95% CI	*p*-Value
Potential confounders			
Maternal age (years)	1.00	0.92–1.08	0.908
Ethnicity: Thai	1.67	0.33–8.36	0.532
Infant age below 6 months	0.71	0.36–1.39	0.315
Family income below USD 16,130 per year	1.43	0.66–3.08	0.361
Education below undergraduate	0.79	0.30–2.09	0.639
Not working/unemployed	0.65	0.15–2.88	0.570
Preventive factors			
Received infant feeding support from health personnel	0.39	0.16–0.94	0.035 *
Enough maternal health support	0.43	0.18–1.02	0.056
Contact with healthcare services	0.46	0.22–0.96	0.040 *
Contact with a “Mother and Baby of breastfeeding support group”	0.95	0.65–1.37	0.778
Risk factors			
Household crowding after lockdown	1.70	0.75–3.87	0.202
Family stress from confinement	1.83	0.82–4.06	0.138
Lack of family support and help with feeding your baby after lockdown	7.04	1.92–25.84	0.003 **

aOR = Adjusted odds ratio, CI = Confidence interval, * Significant association at *p* < 0.05, ** Significant association at *p* < 0.01.

## Data Availability

The data presented in this study are available on request from the correspondent author.
